# A Detailed Spatial Expression Analysis of Wing Phenotypes Reveals Novel Patterns of Odorant Binding Proteins in the Soybean Aphid, *Aphis glycines*

**DOI:** 10.3389/fphys.2021.702973

**Published:** 2021-07-28

**Authors:** Ling Wang, Hang Yin, Zhiguo Zhu, Shuai Yang, Jia Fan

**Affiliations:** ^1^College of Agronomy and Biotechnology, Hebei Normal University of Science and Technology, Qinhuangdao, China; ^2^State Key Laboratory for Biology of Plant Diseases and Insect Pests, Institute of Plant Protection, Chinese Academy of Agricultural Sciences, Beijing, China; ^3^Wuhu Institute of Technology, Wuhu, China

**Keywords:** odorant binding protein, wing phenotype, *Aphis glycines*, expression pattern analysis, cauda, cornicle, antenna, wing

## Abstract

The wide range of insect niches has led to a rapid expansion of chemosensory gene families as well as their relatively independent evolution and a high variation. Previous studies have revealed some functions for odorant-binding proteins (OBPs) in processes beyond olfaction, such as gustation and reproduction. In this study, a comparative transcriptomic analysis strategy was applied for the soybean aphid, *Aphis glycines*, focusing on various functional tissues and organs of winged aphids, including the antenna, head, leg, wing, thorax, cauda, and cornicle. Detailed spatial OBP expression patterns in winged and wingless parthenogenetic aphids were detected by RT-qPCR. Twelve OBPs were identified, and three new *OBP*s in *A. glycines* are first reported. All *OBP*s showed comparatively higher expression in sensory organs and tissues, such as the antenna, head, or leg. Additionally, we found some novel expression patterns for aphid OBPs (Beckendorf et al., 2008). Five OBPs exhibited high-expression levels in the cauda and four in the cornicle (Biasio et al., 2015). Three genes (*OBP2/3/15*) were highly expressed in the wing (Calvello et al., 2003). Two (*OBP3/15*) were significantly more highly expressed in the wingless thorax than in the winged thorax with the wings removed, and these transcripts were significantly enriched in the removed wings. More details regarding OBP spatial expression were revealed under our strategy. These findings supported the existence of carrier transport functions other than for foreign chemicals and therefore broader ligand ranges of aphid OBPs. It is important for understanding how insect OBPs function in chemical perception as well as their other potential physiological functions.

## Introduction

The olfactory system plays an important role in directing insect behaviors, such as foraging, mating, oviposition, and predation. Similar to other insects, aphids, especially those with a migratory phenotype (winged morph), rely heavily on chemical signals, including plant volatiles and species-specific pheromones, to locate hosts, find mates, and avoid natural enemies (Pickett, 2009). In general, odorant-binding proteins (OBPs) are involved in the first step of olfactory recognition (e.g., Pelosi et al., 2018). They bind and transport external odors through the hemolymph to activate corresponding olfactory receptors, which are responsible for transmitting environmental chemicals into electrophysiological signals. As one of the most important groups of chemo-reception proteins in insects, OBPs have been studied since 1981 (Vogt and Riddiford, 1981). Regarding aphids, OBPs have been widely reported for *Acyrthosiphon pisum* (Ji et al., 2016) genes encoding complete OBPs with a signal peptide, Zhou et al., 2010), *Myzus persicae* (Ji et al., 2016), *Megoura viciae* (Iovinella et al., 2011; Daniele et al., 2018), *Sitobion avenae* (Kim et al., 2013; Xue et al., 2016), *Aphis gossypii* (Grabherr et al., 2011; Gu et al., 2013), and *Aphis glycines* (Grabherr et al., 2011; Wang et al., 2019). Consistent with the simple facultative parasitic lifestyle of aphids, the aphid OBP family has few members, and they are generally more highly conserved than in other insects. The spatial expression profiles of these proteins have also been broadly investigated. OBP expression is not limited to the chemosensory system (e.g., Xue et al., 2016; Gao et al., 2018; Wang et al., 2019), but also occurs in non-sensory tissues and organs, such as the wings (Calvello et al., 2003; Pelosi et al., 2005; Wang et al., 2020), reproductive organs (Li et al., 2008; Sun Y. F. et al., 2012), mandibular glands (Iovinella et al., 2011), and salivary glands (Zhang et al., 2017).

The soybean aphid, *A. glycines*, is an important phytophagous pest that feeds on plants by sucking sap from leaves, stems, and pods, significantly reducing soybean yield and quality (Wang et al., 1996; Beckendorf et al., 2008; Wu et al., 2009). Moreover, in soybean plants heavily infested with aphids, sugary excretions (“honeydew”) produced by aphids indirectly damage plants by reducing photosynthesis (Sun et al., 2015). Plant viruses can be transmitted during aphid infestations (Clark and Perry, 2002). Accordingly, *A. glycines* is now used as a model for studying the evolution of biotypic virulence (Wenger et al., 2017). In a recent study, we attempted to identify the OBP family of soybean aphids based on a homologous cloning strategy (Wang et al., 2019). In this study, a more detailed comparative transcriptomic analysis strategy focusing on various functional tissues and organs of winged aphids, including the antenna, head, leg, wing, thorax, cauda, and cornicle, was successfully applied to *A. glycines*. In addition to identifying more OBPs, detailed spatial expression patterns of both winged and wingless parthenogenetic aphids were analyzed by RT-qPCR and the findings were discussed.

## Materials and Methods

### Insects and Sampling

*Aphis glycines* was reared from parthenogenetic aphids initially collected on soybean plants at the Minzhu Experimental Station, Heilongjiang Academy of Agricultural Sciences, China, and cultured in an air-conditioned insectary [24 ± 1°C, 75 ± 5% relative humidity (RH), 16-h light:8-h dark photoperiod]. Newborn aphids (0–12 h) were transferred to new plants for synchronization of developmental stages. The insects were reared for 7 days at different densities (20 aphids/plant and 100 aphis/plant) and winged and wingless aphids were separately collected in the last developmental stage (adult). For transcriptome sequencing, antenna (500), head (200), wing (500), leg (500), thorax (200), cornicle (200), and cauda (500) specimens of winged aphids were carefully dissected under the microscope. For RT-qPCR analysis, the same tissues or organs of adult wingless and winged morphs were collected. Each experiment was carried out in biological triplicate. Samples were flash-frozen in liquid nitrogen and stored at –80°C until RNA extraction.

### Total RNA Extraction and Illumina HiSeq 4000 Sequencing

Total RNA was isolated from the above samples using TRIzol (Invitrogen, Carlsbad, CA, United States), and DNA fragments were removed with RNase-free DNase I (Takara, Shiga, Japan). An Agilent 2100 Bioanalyzer (Agilent Technologies, Carlsbad, CA, United States) was used to determine the concentrations, integrity, and 28S/18S values of the RNA samples, and a NanoDrop 2000 spectrophotometer (NanoDrop products, Wilmington, DE, United States) was used to access purity. mRNA was then enriched using oligo (dT) beads (Agilent Technologies) and fragmented using fragmentation buffer (Agilent Technologies) and then used for the synthesis of first-strand cDNA. After purification and the repair of cohesive ends, the DNA samples were ligated to adapters, and fragment selection and PCR amplification were conducted. The final quality assessment was performed using an Agilent 2100 Bioanalyzer (Agilent Technologies). Three DNA libraries were examined using the Illumina HiSeq 4000 sequencing platform.

Fragmentation involved the use of divalent cations under elevated temperature in NEBNext and first-strand synthesis reaction buffer (5×). Single-stranded (ss) cDNA was synthesized using a random hexamer primer, M-MuLV reverse transcriptase, DNA polymerase I, and RNase H (NEB, United States). After the adenylation of the 3′ ends of the fragments, NEBNext adaptors with a hairpin loop structure were ligated for hybridization. The library fragments were purified using the AMPure XP system (Beckman Coulter, United States), selecting cDNA fragments 150–200 bp long. Then, 3 mL of USER enzyme (NEB, United States) was applied to the size-selected, adaptor-ligated cDNA, and the reaction was incubated at 37°C for 15 min, followed by 5 min at 95°C before PCR. PCR was then performed using Phusion high-fidelity DNA polymerase, universal PCR primers, and an index (X) primer. The products were purified (AMPure XP system), and library quality was assessed using an Agilent Bioanalyzer 2100 system (Agilent Technologies, United States). Clustering of the index-coded samples was performed with a cBot cluster generation system using TruSeq PE Cluster Kit v3-cBot-HS (Illumina, China) according to the manufacturer’s instructions. The library preparations were sequenced on the Illumina HiSeq 4000 platform, and paired-end reads (PE125 sequencing strategy) were generated after cluster generation.

### RNA-Seq Data Generation and *de novo* Transcriptome Assembly

After sequencing, the raw reads were processed by NGS-QC to remove low-quality sequences (≥ 15% bases with Q ≤ 19), excess adaptors (≥ 5 bp adaptor bases in reads), and reads with a high content of unknown bases (≥ 5%; CASAVA FASTQ files). The clean reads were then assembled into unigenes using Trinity r20140413p1 with min_kmer_cov:2 and the other parameters set to the default values (Langmead and Salzberg, 2012). Gene expression levels in each sample were estimated by RSEM (Li and Dewey, 2011): (I) Clean data were mapped to the transcript sequence, and (II) the read count for each gene and isoform was obtained from the mapping results. The fragments per kilo base per million (FPKM)-mapped reads value of each gene was calculated based on gene length and the mapped read number using HTSeq v0.5.4p3 and Cufflinks v2.2.1 (Mortazavi et al., 2008).

### Differentially Expressed Genes and Annotation of OBP-Encoding Transcripts

Reads for the *A. glycines* transcriptomes from seven different tissues (antennae, head, wing, leg, thorax, cornicle, and cauda), with three replications for each tissue, were produced based on next-generation sequencing (NGS) results. Expression analysis was performed using TopHat and Cufflinks (Trapnell et al., 2012; Kim et al., 2013). Differential expression analyses comparing each tissue to the antenna were separately performed using the DESeq R package (version 1.10.1), which provides statistical routines for determining differential expression using a model based on the negative binomial distribution. To control the false discovery rate, the resulting *p*-values were adjusted using Benjamini and Hochberg’s approach. Genes with a fold change (FC) > 2 and an adjusted *p*-value < 0.05 according to DESeq analysis were considered DEGs. The log2 (fold change) values and *p* values are shown in a volcano plot.

We used the BLASTx program of the National Center for Biotechnology Information (NCBI,^1^) to predict genes-encoding OBPs.

The basis of the annotation was a hand-curated database of OBPs containing known aphid candidate OBP sequences. The assembled sequences were compared with the reference dataset using BLASTx. All sequences that generated a hit were further analyzed by a motif search program based on a 5–6 conserved OBP cysteine pattern consisting of C1-X_15–39_-C_2_-X_3_-C_3_-X_21–44_- C_4_-X_7–12_-C_5_-X_8_-C_6_ for OBPs (Zhou et al., 2008).

### Functional Annotation Enrichment Analysis

According to the DEG results, Venn diagrams of the differentially expressed olfaction genes in group 1 (antennae/head), group 2 (antennae/leg), group 3 (antennae/wing), group 4 (antennae/thorax), group 5 (antennae/cornicle), and group 6 (antennae/cauda) were constructed using “Venn Diagram”^2^. The mean FPKM values for each gene in the different tissues (antenna, head, leg, wing, thorax, cornicle, and cauda) were then log-transformed [“log2 (FPKM + 1)”] and subjected to hierarchical clustering using the minimum spanning tree; a heatmap was generated using Heml1.0 (Deng et al., 2014). Antenna-specific genes were defined as DEGs identified in tissues other than antennae with FPKM ≤ 0.3 (e.g., Sánchez-Sevilla et al., 2017; Tao et al., 2017).

### Real-Time Quantitative PCR

Real-time quantitative PCR (RT-qPCR) experiments were carried out using a 7,500 Fast Real-Time PCR System (Applied Biosystems- Life Technologies, Carlsbad, CA, United States) and the cDNA samples prepared from winged and wingless aphid antennae, heads, legs, cornicles, caudae, and wings (only for winged morphs). Two reference genes, 18S *rRNA* and GAPDH dehydrogenase, were used for normalizing target gene expression and correction for sample-to-sample variation (Wang et al., 2019). Specific primers were designed for each *A. glycines* OBP gene and for the two reference genes using Primer Premier v5.0 software; the primer information is listed in Supplementary Data 1. PCR amplification was conducted in a volume of 20 μL containing 10 μL of 2 × SYBR Mix, 1 μL of diluted cDNA template, 7.8 μL of PCR-grade water, and 0.6 μL of each primer at 10 μM. The PCR conditions were as follows: 95°C for 30 s, 40 cycles of 95°C for 15 s, 60°C for 30 s, and 72°C for 45 s. The OBP expression status was calculated using the 2^–ΔΔCt^ comparative CT method (Livak and Schmittgen, 2001), and a CT value greater than 35 was considered no expression. The fold changes of *OBP*s in the tissues of both winged and wingless morphs are reported relative to the antennal transcript levels of *OBP3* in the wingless morph (wingless antennal *OBP3*). Means and standard deviations were calculated using data from experiments performed in triplicate, and the results were presented as *n*-fold differences in expression. Differences in transcriptional characteristics among various *OBP*s in different tissues were analyzed using SPSS 16.0. Statistical significance was determined using one-way ANOVA and *post hoc* Duncan multiple range tests. Significance was established at *p* < 0.05. External reference genes were randomly chosen from among *OBP*s to first perform a preliminary assessment, after which, we defined those with broader expression profiles, such as wingless *OBP2* and *OBP3*, as candidate external genes. Wingless *OBP*3 was ultimately chosen as the external gene.

## Results

### Overview of Transcriptomes

To identify and differentiate *OBP*s transcripts among antennae, heads, legs, wings, thoraxes, cornicles, and caudae, 18 mRNA samples from the 7 different tissues (each analyzed in triplicate) were subjected to 2 × 125 paired-end sequencing using the HiSeq 4000 platform, yielding 167,359,594 bases. A total of 154,717 distinct transcripts (mean length = 1,082 bp) and 110,897 unigenes (mean length = 629 bp) were assembled (Supplementary Data 2).

**FIGURE 1:**
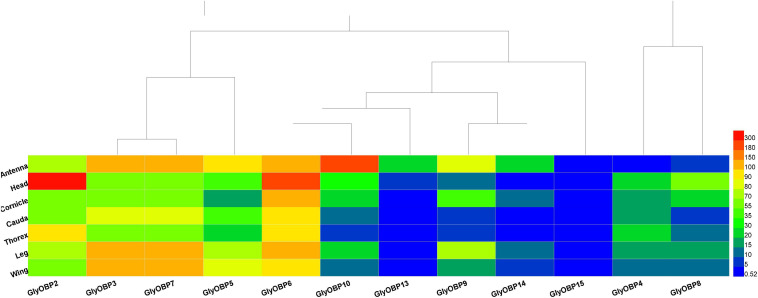
Volcano plots for differentially expressed genes between antennae and each of the other six tissues (heads, legs, caudae, cornicles, thoraxes, and wings).

Gene expression analysis showed the following numbers of DEGs with a log2-fold change ≥ 2 (*p*_*adj*_ value ≤ 0.05) in each paired comparison group. Compared with the antenna, the thorax showed the 14,430 significantly differentially expressed genes (antenna/thorax, 2,565 upregulated and 11,865 downregulated). The antenna/leg value was 14,979 (5,781 upregulated and 9,198 downregulated), the antenna/head value 13,040 (2,757 upregulated and 10,283 downregulated), the antenna/cauda value 12,549 (3,797 upregulated and 8,752 downregulated), the antenna/cornicle value 11,537 (3,801 upregulated and 11,456 downregulated), and antenna/wing value 12,719 (1,267 upregulated and 10,912 downregulated) (Figure 1 and Supplementary Data 3).

### Differential Expression Analysis

**FIGURE 2:**
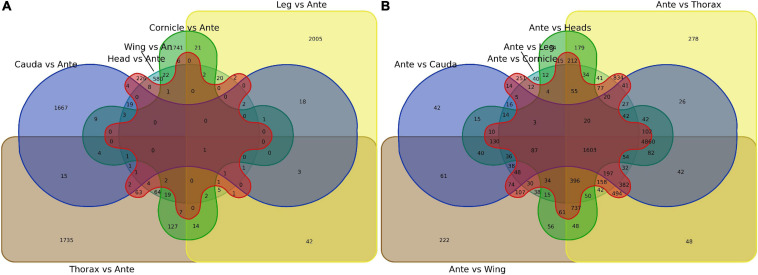
Venn diagram of specially expressed transcripts between antennae and six other tissues (heads, legs, caudae, cornicles, thoraxes, and wings). **(A)** Specially expressed transcript numbers for six tissues compared with antennae. **(B)** Specially expressed transcript numbers for antennae compared with each of the other six tissues. An or Ante, Antenna.

First, antenna-specific genes were compared among different groups and further analyzed using Venn diagrams. Although genes were found to exhibit antenna specific expression, there were no *OBP*s (Figure 2A; gene lists see Supplementary Data 3). Next, genes specifically expressed in each tissue were screened by the same strategy, and the numbers showing specific expression in the heads, legs, wings, thoraxes, caudae, and cornicles were 226, 2,005, 580, 1,735, 1,667, and 1,741, respectively, also with no *OBP*s included (Figure 2B, see Supplementary Data 3 for gene lists).

### OBP Prediction and Functional Enrichment

Odorant-binding proteins genes were predicted by homology comparison, the relative expression levels (FPKM) of target genes in different tissues were visualized by a heat map, and the tissue or organ specificity of expression was preliminarily analyzed.

**FIGURE 3:**
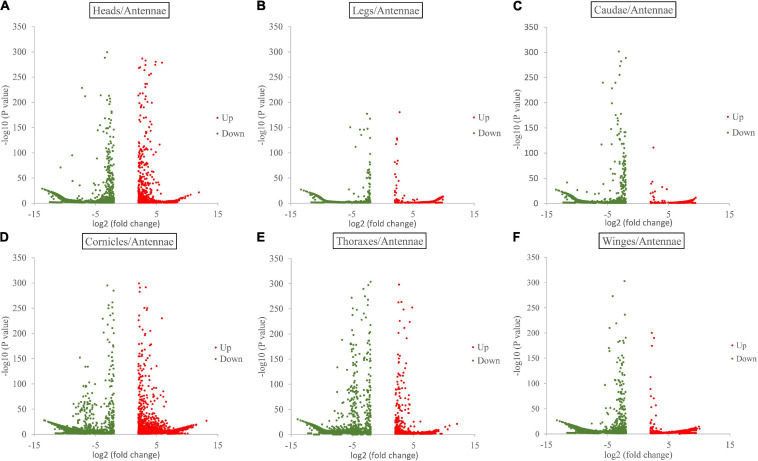
Heatmap of 12 *OBPs* (*OBP2-10*, *OBP13-15*) FPKM values from the transcriptomes of 7 tissues and organs (antennae, heads, wings, legs, thoraxes, caudae, and cornicles). **(A–F)** were the heatmap result of 12 *OBPs* FPKM values in heads, legs, caudae, cornicles, thoraxes, and wings, respectively.

Similar to peach aphids (Ji et al., 2016), *OBP1* was neither predicted nor identified in *A. glycines*. Twelve OBPs were identified (GenBank accession numbers MW924836-MW924846, and MW930727) using the NCBI BLASTX program and named according to their ortholog names in other aphids, including three newly reported *OBP*s: *OBP13*, *OBP14*, and *OBP15*. The *OBP*s included in the heatmap and the sequences of these genes are listed in Supplementary Data 4; their FKPM values are provided in Supplementary Data 5. The heat map in Figure 3 illustrates that most of the *OBP*s, such as *OBP2-*OBP10, *OBP13*, and *OBP14*, were found to mainly be expressed in sensory organs and tissues (i.e., antennae, heads and legs) but that OBP15 showed relatively low expression in all specimen types. Our results showed that *OBP*s are also expressed in organs such as caudae and cornicles, which are not chemical sensory organs. As *OBP3* and *OBP7* were assembled into one transcript (DN1170_c0_g1_i8, Supplementary Data 4) following the TRINITY instructions (Grabherr et al., 2011), their gene expression values were ultimately quantified as equal and then were corrected by RT-qPCR.

### Detailed Spatial Expression Analysis Between Morphs by RT-qPCR

To investigate tissue expression specificity and further verify any phenotypic correlations, the spatial expression profiles of OBPs in both winged and wingless aphids were detected by RT-qPCR.

**FIGURE 4:**
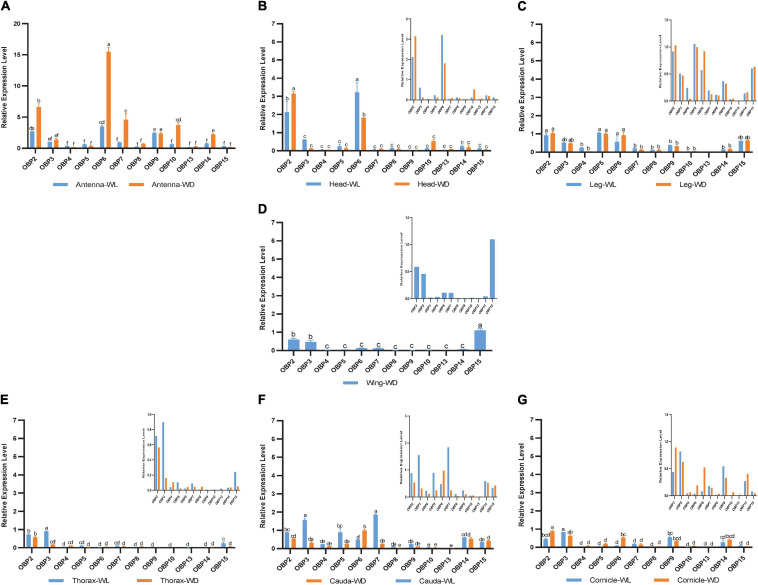
RT-qPCR detection of 12 OBP expression patterns at the mRNA level in each tissue. WL, wingless; WD, winged; bars represent the standard deviation of the mean for three independent experiments. **(A–G)** are the results of 12 OBP expression patterns in antennae, heads, legs, wings, thoraxes, caudae, and cornicles, respectively. The letters above bars (a–f) are the result of a multicomparison, which indicated significant differences from other samples with different letters (*p* < 0.05).

**FIGURE 5:**
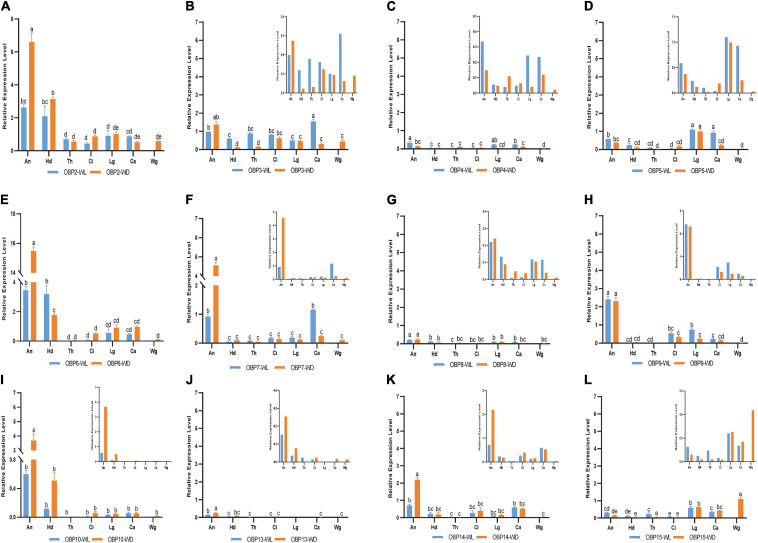
RT-qPCR detection of each OBP expression pattern in seven tissues; WL, wingless; WD, winged; An, antennae; Hd, head; Th, thorax; Ci, cornicle; Lg, leg; Ca, cauda; Wg, wing. Bars represent the standard deviation of the mean for three independent experiments. **(A–L)** are the detection results of each OBP expression pattern in seven tissues and the sequence is from OBP2 to OBP10 and OBP13 to OBP15, respectively. The letters above bars (a–e) are the result of a multicomparison, which indicated significant differences from other samples with different letters (*p* < 0.05).

According to this analysis (Figure 4), 12 *OBP*s are highly expressed in sensory organs such as the antenna, head, and leg. Among these *OBP*s, six *OBP*s (OBP2/6/7/9/10/14) showed the highest transcript levels in antennae (Figure 4A, *p* < 0.05, *N* = 3). Moreover, *OBP4/8/13* exhibited comparatively higher expression in antennae than in other tissues (Figures 5C,G,J, *p* < 0.05, *N* = 3), although the levels were relatively low. In summary, nine *OBP*s (*OBP2/4/6/7/8/9/10/13/14*) were more highly expressed in antennae than in other tissues. Furthermore, seven (*OBP2/6/7/8/10/13/14*) of the nine OBPs mentioned above were significantly more highly expressed in the antennae of winged aphids than in wingless aphids; in contrast, *OBP*4 showed wingless antenna-specific expression, and *OBP*9 was highly expressed, but without a difference between winged and wingless antennae (Figure 5, *p* < 0.05, *N* = 3).

In addition to its remarkably higher expression in winged antennae, *OBP*2 was found to be systemically expressed in all tissues of both morphs, including the antenna, head, leg, wing, thorax, cornicle, and cauda (Figure 5A). *OBP3* was also systemically expressed, with significantly higher expression in the wingless head, thorax, and cauda, and therefore showed a winged aphid-specific expression pattern in those tissues (Figure 5B, *p* < 0.05, *N* = 3). *OBP*4 was expressed at quite a low level but still showed a phenotypic correlation with wingless aphids, with comparatively high expression in the antenna, leg, and cauda. *OBP*5 was leg specific in both morphs, whereas *OBP*15 was highly expressed in the wings and legs of both winged and wingless morphs (Figure 5).

In addition to *OBP*2, *OBP*6 maintained high expression levels in the head of winged and wingless aphids (Figure 4B). Specifically, *OBP*2 was more highly expressed in the winged aphids head and *OBP*6 in the wingless aphid head.

Similar to *OBP*3, *OBP*15 was more highly expressed in the wingless aphids thorax. *OBP*2 was highly expressed in both winged and wingless morphs.

In the leg, the expression of most OBPs was quite low and showed no phenotypic correlation. Among them, *OBP2/5/6* displayed comparatively high expression, with *OBP*5 being significantly more highly expressed in the leg than in other tissues (Figure 4C).

Surprisingly, we found that three *OBP*s, *OBP2*, *OBP3*, and *OBP15*, were expressed at much higher levels in the wing than other OBPs (Figure 4D).

The results for the cornicle indicated that five OBPs (*OBP2/3/6/9/14*) were highly expressed; among them, *OBP2/6* showed differential expression between morphs, though they all presented significantly elevated expression in the winged morph. Nevertheless, the expression of the other three OBPs (*OBP3/9/14*) did not differ between morphs. In addition to *OBP3* and *OBP9*, the gene coding the other EBF-binding protein, *OBP7*, was expressed in the cornicle at relatively low expression levels, with no significant difference between winged and wingless morphs.

*OBP3/5/7* generally appeared to be specific to the wingless morph cauda. *OBP6* was highly expressed in wingless aphids; *OBP2* was also highly expressed, but with no significant difference between winged and wingless aphids.

## Discussion

In this study, three newly reported OBPs were identified based on TRINITY, which has been demonstrated to recover more full-length transcripts across a broad range of expression levels, with a sensitivity similar to the methods that rely on genome alignments (Grabherr et al., 2011). Our differential expression analysis (Figure 1) and enrichment results (Figure 3) showed that OBPs are widely expressed in the soybean aphid, although none was found to be antenna specific or specific to any of seven organs/tissues (Figure 2). The results derived from the aforesaid transcriptome data prompted us to further carry out a detailed investigation and analysis of *OBP* expression levels among different wing phenotypes and different tissue parts based on RT-qPCR technology. The qPCR results show that relatively high expression of most OBPs in antennae, the main olfaction organ of aphids, of both phenotypes, and further, higher expression in the winged phenotype (Figure 4) are consistent with the fact that winged aphids have more developed olfaction (Pickett, 2009) and support that OBPs play key roles in aphids’ olfaction.

Our work provides insight into the potential functions of OBPs correlating with their spatial expression among seven body parts, including various functional organs and tissues, including the antenna, head, leg, thorax, wing, cauda, and cornicle. We further report the breakthrough of the acquisition of aphid cauda and cornicle transcriptomes and their publication. The insect OBP family is believed to participate in chemosensory perception due to their high abundance in chemosensory organs, such as antennae, heads, and legs (e.g., Vogt and Riddiford, 1981; Sun et al., 2013). Without exception, all 12 OBPs identified in this study exhibited relatively high expression in the antenna, head, or leg of both *A. glycine* wing morphs. Most *OBPs* showed relatively high expression in the antennae, the main olfaction organ of aphids, in both phenotypes, with higher expression in the winged phenotype (Figure 4), consistent with the fact that winged aphids have more developed olfaction (Pickett, 2009).

OBP3 (Qiao et al., 2009), OBP7 (Sun Y. L. et al., 2012; Zhong et al., 2012; Fan et al., 2017), and OBP9 (Qin et al., 2020) in aphids are known for their high affinities for E-β-farnesene, the key component of the aphid alarm pheromone. In this study, the genes encoding the three EBF-binding proteins (*OBP3/7/9*) showed different antennal expression patterns from each other (Figure 5), providing a new perspective for understanding relationships among them. *OBP7* was significantly highly expressed in the antenna of both phenotypes, with higher levels in winged aphids. In contrast, there was no difference in the expression of *OBP3* or *OBP9* between the two phenotypes. Further analysis showed that *OBP3* was systemically expressed; significantly higher expression in the head, thorax, and cauda of wingless aphids was detected. However, *OBP9* presented with high expression in the antenna, leg, and cornicle. The higher expression level of *OBP7* in the antennae of winged aphids suggests that it may contribute more to the EBF sensitivity of winged aphids. Notably, the genes encoding these three reported EBF-binding proteins were all expressed in the cornicle (Figure 4G). Cornicles comprising a pair of tubular tissues involved are the alarm pheromone (E-β-farnesene, EBF) storage and release organ, and *OBP3*, *OBP7*, and *OBP9* may be involved in alarm pheromone activity preservation, release or biosynthesis, and metabolism by binding to and releasing EBF.

Insect OBPs have been reported to act as carrier proteins in the male reproductive apparatus of mosquitoes (Li et al., 2008). After matting, the OBP expressed by male moths is found on the surface of fertilized eggs, which helped the larvae to avoid cannibalistic behaviors (Sun Y. L. et al., 2012). In this study, caudae were dissected with dorsal segments of the distal and abdominal segments, anus, and gonapophysis. Therefore, the high expression of OBPs observed in this tissue suggests potential functions in reproduction or excretion. In addition, carrier proteins function in the binding of or transfer of foreign chemicals or signal ligands.

Odor-binding proteins have also been found to be expressed in wings, such as in *Polistes dominula* (Calvello et al., 2003), *Vespa crabro* (Pelosi et al., 2005), and *Helicoverpa armigera* (Wang et al., 2020). Wang et al. further demonstrated lipid binding by OBPs indicating roles beyond their typical functions in the olfactory system to support insect flight activity. In this study, two *OBP*s (*OBP3* and *OBP15*) were found to be expressed in the thorax of the wingless phenotype and were significantly downregulated in the winged thorax with wings removed (*p* < 0.05, Figures 4D,E, 5B,L). In addition, the removed wings expressed significantly high levels of both. Hence, there is a possibility that *OBP3* and *OBP15* were enriched from the thorax to the wings and that they may also function in other ways, such as lipid-binding proteins in the energy supply of flight or carrier proteins which we discussed above in the section on caudae.

Although *OBP3/7/9* all exhibit an affinity for EBF, they showed differential expression patterns in this study. *OBP3* was extensively expressed throughout the aphid body, *OBP7* was antenna-specifically expressed, and *OBP9* was highly expressed in the antenna, leg, and cauda. As the cornicle is the alarm pheromone (E-β-farnesene, EBF) storage and release organ, it was not surprising to find that all previously reported EBF-binding proteins were expressed in the cauda (*OBP3*, *OBP9*, and low expression of *OBP7*).

*SaveOBP2*, *SaveOBP4*, and *SaveOBP5* have been reported to have a limited affinity for wheat volatile benzaldehyde (Zhong et al., 2012). However, no potential ligand has yet been reported for *OBP6*, one of the most highly expressed OBPs, which suggests that the ligand spectrum for insect OBPs may be far greater than our expectations.

More details regarding OBP spatial expression were revealed under our strategy. These findings supported the existence of carrier transport functions other than for foreign chemicals and therefore broader ligand ranges of aphid OBPs. It is important for understanding how insect OBPs function in chemical perception as well as other physiological functions of OBPs.

## Data Availability Statement

The transcriptomic datasets presented in this study can be found in online repositories (http://www.ncbi.nlm.nih.gov/bioproject/744282) with temporary submission ID: SUB9958208.

## Author Contributions

LW and JF conceived and designed the study. LW and SY performed the experiments. HY and JF assembled and analyzed the transcriptome data. SY analyzed the RT-qPCR data analysis. JF, SY, and LW wrote the original manuscript. All the other authors contributed on polishing and revising the article.

## Conflict of Interest

The authors declare that the research was conducted in the absence of any commercial or financial relationships that could be construed as a potential conflict of interest.

## Publisher’s Note

All claims expressed in this article are solely those of the authors and do not necessarily represent those of their affiliated organizations, or those of the publisher, the editors and the reviewers. Any product that may be evaluated in this article, or claim that may be made by its manufacturer, is not guaranteed or endorsed by the publisher.

## Footnotes

1
http://blast.ncbi.nlm.nih.gov/Blast.cgi

2
http://bioinformatics.psb.ugent.be/webtools/Venn/

## Supplementary Material

Supplementary Data 1 RT-qPCR primer list.

Supplementary Data 2 Transcriptome data information.

Supplementary Data 3 Differential expression transcript list.

Supplementary Data 4 GlyOBP sequences.

Supplementary Data 5 GlyOBP FPKM value.

## References

[B1] Beckendorf E. A. Catangui M. A. Riedell W. E. (2008). Soybean aphid feeding injury and soybean yield, yield components, and seed composition. *Agron. J.* 100 237–246. 10.2134/agrojnl2007.0207

[B2] Biasio F. D. Riviello L. Bruno D. Annalisa G. Congiu T. Sun Y. F. (2015). Expression pattern analysis of odorant-binding proteins in the pea aphid *Acyrthosiphon pisum*. *Insect Sci.* 22 220–234. 24591440 10.1111/1744-7917.12118

[B3] Calvello M. Guerra N. Brandazza A. D’Ambrosio C. Scaloni A. Dani F. R. (2003). Soluble proteins of chemical communication in the social wasp Polistes dominulus. *Cell. Mol. Life Sci.* 60 1933–1943. 10.1007/s00018-003-3186-5 14523553 PMC11138633

[B4] Clark A. Perry K. L. (2002). Transmissibility of field isolates of soybean viruses by *Aphis glycines*. *Plant Dis.* 86 1219–1222. 10.1094/PDIS.2002.86.11.1219 30818470

[B5] Daniele B. Gerarda G. Rosanna S. Andrea S. Donatella F. Annalisa G. (2018). Sensilla morphology and complex expression pattern of odorant binding proteins in the vetch aphid *Megoura viciae* (hemiptera: aphididae). *Front. Physiol.* 9:777. 10.3389/fphys.2018.00777 29988577 PMC6027062

[B6] Deng W. Wang Y. Liu Z. Cheng H. Xue Y. (2014). HemI: a toolkit for illustrating heatmaps. *PLoS One* 9:e111988. 10.1371/journal.pone.0111988 25372567 PMC4221433

[B7] Fan J. Xue W. Duan H. Jiang X. Zhang Y. Yu W. (2017). Identification of an intraspecific alarm pheromone and two conserved odorant-binding proteins associated with (E)-β-farnesene perception in aphid *Rhopalosiphum padi*. *J. Insect Physiol.* 101 151–160. 28778653 10.1016/j.jinsphys.2017.07.014

[B8] Gao K. X. Zhang S. Luo J. Y. Wang C. Y. Li-Min L. Zhang L. J. (2018). Molecular characterization and ligand-binding properties of six odorant binding proteins (OBPs) from Aphis gossypii. *J. Asia Pac. Entomol.* 21 914–925. 10.1016/j.aspen.2018.07.004

[B9] Grabherr M. G. Haas B. J. Yassour M. Levin J. Z. Thompson D. A. Amit I. (2011). Full-length transcriptome assembly from RNA-Seq data without a reference genome. *Nat. Biotechnol.* 29 644–652. 10.1038/nbt.1883 21572440 PMC3571712

[B10] Gu S. H. Wu K. M. Guo Y. Y. Field L. M. Pickett J. A. Zhang Y. J. (2013). Identification and expression profiling of odorant binding proteins and chemosensory proteins between two wingless morphs and a winged morph of the cotton aphid *Aphis gossypii* glover. *PLoS One* 8:e73524. 10.1371/journal.pone.0073524 24073197 PMC3779235

[B11] Iovinella I. Dani F. R. Niccolini A. Sagona S. Michelucci E. Gazzano A. (2011). Differential expression of odorant-binding proteins in the mandibular glands of the honey bee according to caste and age. *J. Proteome Res.* 10 3439–3449. 10.1021/pr2000754 21707107

[B12] Ji R. Wang Y. Cheng Y. Zhang M. Zhang H. B. Zhu L. (2016). Transcriptome analysis of green peach aphid (*Myzus persicae*): insight into developmental regulation and inter-species divergence. *Front. Plant Sci.* 7:1562. 10.3389/fpls.2016.01562 27812361 PMC5072348

[B13] Kim D. Pertea G. Trapnell C. Pimentel H. Kelley R. Salzberg S. L. (2013). TopHat2: accurate alignment of transcriptomes in the presence of insertions, deletions and gene fusions. *Genome Biol.* 14:R36. 10.1186/gb-2013-14-4-r36 23618408 PMC4053844

[B14] Langmead B. Salzberg S. L. (2012). Fast gapped-read alignment with Bowtie 2. *Nat. Methods* 9 357–359. 10.1038/nmeth.1923 22388286 PMC3322381

[B15] Li B. Dewey C. N. (2011). RSEM: accurate transcript quantification from RNA-Seq data with or without a reference genome. *BMC Bioinform.* 12:323. 10.1186/1471-2105-12-323 21816040 PMC3163565

[B16] Li S. Picimbon J. F. Ji S. Kan Y. Pelosi P. (2008). Multiple functions of an odorant-binding protein in the mosquito *Aedes aegypti*. *Biochem. Biophys. Res. Commun.* 372 464–468. 10.1016/j.bbrc.2008.05.064 18502197

[B17] Livak K. J. Schmittgen T. D. (2001). Analysis of temporal gene expression data using real-time quantitative PCR and the 2 (-Delta Delta C (T)) method. *Methods* 25 402–408. 10.1006/meth.2001 11846609

[B18] Mortazavi A. Williams B. A. Mccue K. Schaeffer L. Wold B. (2008). Mapping and quantifying mammalian transcriptomes by RNA-Seq. *Nat. Methods* 5 621–628. 10.1038/nmeth.1226 18516045 PMC13303166

[B19] Pelosi P. Iovinella I. Zhu J. Wang G. Dani F. R. (2018). Beyond chemoreception: diverse tasks of soluble olfactory proteins in insects. *Biol. Rev*. 93 184–200. 10.1111/brv.12339 28480618

[B20] Pelosi P. Mariantonietta C. Ban L. (2005). Diversity of odorant-binding proteins and chemosensory proteins in insects. *Chem. Senses* 30:i291. 10.1093/chemse/bjh229 15738163

[B21] Pickett J. A. (2009). High-throughput ESI-MS analysis of binding between the *Bombyx mori* pheromone-binding protein BmorPBP1, its pheromone components and some analogues. *Chem. Commun.* 38 5725–5727. 10.1039/b914294k 19774249

[B22] Qiao H. L. Tuccori E. He X. L. Gazzano A. Field L. Zhou J. J. (2009). Discrimination of alarm pheromone (E)–β-farnesene by aphid odorant-binding proteins. *Insect Biochem. Mol. Biol.* 39 414–419. 19328854 10.1016/j.ibmb.2009.03.004

[B23] Qin Y. G. Yang Z. K. Song D. L. Wang Q. Gu S. H. Li W. H. (2020). Bioactivities of synthetic salicylate-substituted carboxyl (E)-β-Farnesene derivatives as ecofriendly agrochemicals and their binding mechanism with potential targets in aphid olfactory system. *Soc. Chem. Ind.* 76 2465–2472. 10.1002/ps.5787 32061021

[B24] Sánchez-Sevilla J. F. Vallarino J. G. Osorio S. Bombarely A. Posé D. Merchante C. (2017). Gene expression atlas of fruit ripening and transcriptome assembly from RNA-seq data in octoploid strawberry (Fragaria × ananassa). *Sci. Rep.* 7:13737. 10.1038/s41598-017-14239-6 29062051 PMC5653846

[B25] Sun W. P. Hu Z. F. Han L. L. Sanda N. B. Xuan Y. H. Zhao K. J. (2015). Discovery of a transitional host of the soybean aphid, *Aphis glycines* (Hemiptera: Aphididae), in northeastern China. *Appl. Entomol. Zool.* 50 361–369. 10.1007/s13355-015-0343-x

[B26] Sun Y. F. De Biasio F. Qiao H. L. Iovinella I. Yang S. X. Ling Y. (2012). Two odorant-binding proteins mediate the behavioural response of aphids to the alarm pheromone (E)-β-farnesene and structural analogues. *PLoS One* 7:e32759. 10.1371/journal.pone.0032759 22427877 PMC3299684

[B27] Sun Y. L. Huang L. Q. Pelosi P. Wang C. Z. (2012). Expression in antennae and reproductive organs suggests a dual role of an odorant-binding protein in two sibling *Helicoverpa* Species. *PLoS One* 7:e30040. 10.1371/journal.pone.0030040 22291900 PMC3264552

[B28] Sun Y. P. Zhao L. J. Sun L. Zhang S. G. Ban L. P. (2013). Immunolocalization of odorant-binding proteins on antennal chemosensilla of the peach aphid *Myzus persicae* (Sulzer). *Chem. Senses* 38 129–136. 10.1093/chemse/bjs093 23222972

[B29] Tao S. Q. Cao B. Tian C. M. Liang Y. M. (2017). Comparative transcriptome analysis and identification of candidate effectors in two related rust species (*Gymnosporangium yamadae* and *Gymnosporangium asiaticum*). *BMC Genomics* 18:651. 10.1186/s12864-017-4059-x 28830353 PMC5567642

[B30] Trapnell C. Roberts A. Goff L. Pertea G. Kim D. Kelley D. R. (2012). Differential gene and transcript expression analysis of RNA-seq experiments with TopHat and Cufflinks. *Nat. Protoc.* 7 562–578. 10.1038/nprot.2012.016 22383036 PMC3334321

[B31] Vogt R. G. Riddiford L. M. (1981). Pheromone binding and inactivation by moth antennae. *Nature* 293 161–163. 10.1038/293161a0 18074618

[B32] Wang L. Bi Y. Liu M. Li W. Liu M. Di S. F. (2019). Identification and expression profiles analysis of odorant-binding proteins in soybean aphid, *Aphis glycines* (hemiptera: aphididae). *Insect Sci.* 27 1019–1030. 10.1111/1744-7917.12709 31271503

[B33] Wang S. Minter M. Homem R. A. Michaelson L. V. Venthur H. Lim K. S. (2020). Odorant binding proteins promote flight activity in the migratory insect, *Helicoverpa armigera*. *Mol. Ecol.* 29 3795–3808. 10.1111/mec.15556 32681685

[B34] Wang S. Y. Bao X. Z. Sun Y. J. Chen R. L. Zhai B. P. (1996). Study on the effect of population dynamics of soybean aphid (*Aphis glycines*) on both growth and yield of soybean. *Soybean Sci. (China)* 15 243–247.

[B35] Wenger J. A. Cassone B. J. Legeai F. Johnston J. S. Bansal R. Yates A. D. (2017). Whole genome sequence of the soybean aphid, *Aphis glycines*. *Insect Biochem. Mol. Biol.* 123:102917. 10.1016/j.ibmb.2017.01.005 28119199

[B36] Wu T. L. Ma X. H. Yao L. M. Wang B. (2009). Identification of Soybean resources of resistance to aphids. *Agric. Sci. China* 8 979–984. 10.1016/S1671-2927(08)60303-X

[B37] Xue W. Jia F. Zhang Y. Xu Q. Han Z. Sun J. (2016). Identification and expression analysis of candidate odorant-binding protein and chemosensory protein genes by antennal transcriptome of *Sitobion avenae*. *PLoS One* 11:e0161839. 10.1371/journal.pone.0161839 27561107 PMC4999175

[B38] Zhang Y. Fan J. Sun J. Francis F. Chen J. (2017). Transcriptome analysis of the salivary glands of the grain aphid, *Sitobion avenae*. *Sci. Rep.* 7:15911. 10.1038/s41598-017-16092-z 29162876 PMC5698471

[B39] Zhong T. Yin J. Deng S. Li K. Cao Y. (2012). Fluorescence competition assay for the assessment of green leaf volatiles and trans-β-farnesene bound to three odorant-binding proteins in the wheat aphid *Sitobion avenae* (Fabricius). *J. Insect Physiol.* 58 771–781. 10.1016/j.jinsphys.2012.01.011 22306433

[B40] Zhou J. J. He X. L. Pickett J. A. Field L. M. (2008). Identification of odorant-binding proteins of the yellow fever mosquito *Aedesa egypti*: genome annotation and comparative analyses. *Insect Mol. Biol.* 17 147–163. 10.1111/j.1365-2583.2007.00789.x 18353104

[B41] Zhou J. J. Vieira F. G. He X. L. Smadja C. Liu R. Rozas J. (2010). Genome annotation and comparative analyses of the odorant-binding proteins and chemosensory proteins in the pea aphid *Acyrthosiphon pisum*. *Insect Mol. Biol.* 19 113–122. 10.1111/j.1365-2583.2009.00919.x 20482644

