# Supplementary material for: A Detailed Spatial Expression Analysis of Wing Phenotypes Reveals Novel Patterns of Odorant Binding Proteins in the Soybean Aphid, Aphis glycines
Source: Front Physiol. 2021 Jul 28;12:702973. doi: 10.3389/fphys.2021.702973 (PMC8376974; doi:10.3389/fphys.2021.702973)
Supplement: Supplementary Data 1 — RT-qPCR primer list. [file Table_1.docx]

Table S1 Primers used for RT-qPCR.

| Primer name | Sequences of primers |
| --- | --- |
| AglyOBP2-F | ATGAAGGTATCTGCAGCGACC |
| AglyOBP2-R | GTCGAACTGTGCACATGGTCC |
| AglyOBP3-F | CGCTGATGTTTGGTATTGCAATG |
| AglyOBP3-R | GCAGTCCTAGTTTGCTGATCATGC |
| AglyOBP4-F | GTGTTTGCTCCAGTGCGTCTAC |
| AglyOBP4-R | GCAAGATCACACGTTTGTCCATC |
| AglyOBP5-F | GTGCAACGATGAAATGCGTC |
| AglyOBP5-R | GCGTAACACTCGTCAGCTGCTAC |
| AglyOBP6-F | CCTACATGTTGTCAAATGCCA |
| AglyOBP6-R | TCGCTAGTAGTTAATCCAGATTGC |
| AglyOBP7-F | CTCGCCGTTGTAGCAGCAAC |
| AglyOBP7-R | GTGCCAACATCGTCATCTTGTC |
| AglyOBP8-F | GTCGGTCGCTGTCGTATTCG |
| AglyOBP8-R | GTGCCACTTTAGTTGCCTCTTC |
| AglyOBP9-F | GATGCAGATACAGCGGATAAGG |
| AglyOBP9-R | CACACGCCATGTGACATGTAC |
| AglyOBP10-F | GGACACTCACCCTGATGTGACC |
| AglyOBP10-R | TGGTTGTGGTCGTGTAGATGAC |
| AglyOBP13-F | GCGAGTTCAGCGTAAACCGAC |
| AglyOBP13-R | TGCGTCGTAATCGACACTTGG |
| AglyOBP14-F | CGAAGAGAATGGGAACGATG |
| AglyOBP14-R | GCAGTGAGTGTTTACGTCATCG |
| AglyOBP15-F | GGAGGTTACAATGATAAGGCATTG |
| AglyOBP15-R | GTCGTCGACGATCAGCTGAC |
| GAPDH-F | CAAGGGCGAAGTCTCTGTTGA |
| GAPDH-R | CTACTACGTAATCAGCGCCAGC |
| 18S rRNA-F | ATTGACGGAAGGGCACC |
| 18S rRNA-R | CGCTCCACCAACTAAGAACG |
