# Supplementary figures and images for: A Detailed Spatial Expression Analysis of Wing Phenotypes Reveals Novel Patterns of Odorant Binding Proteins in the Soybean Aphid, Aphis glycines
Source: Front Physiol. 2021 Jul 28;12:702973. doi: 10.3389/fphys.2021.702973 (PMC8376974; doi:10.3389/fphys.2021.702973)

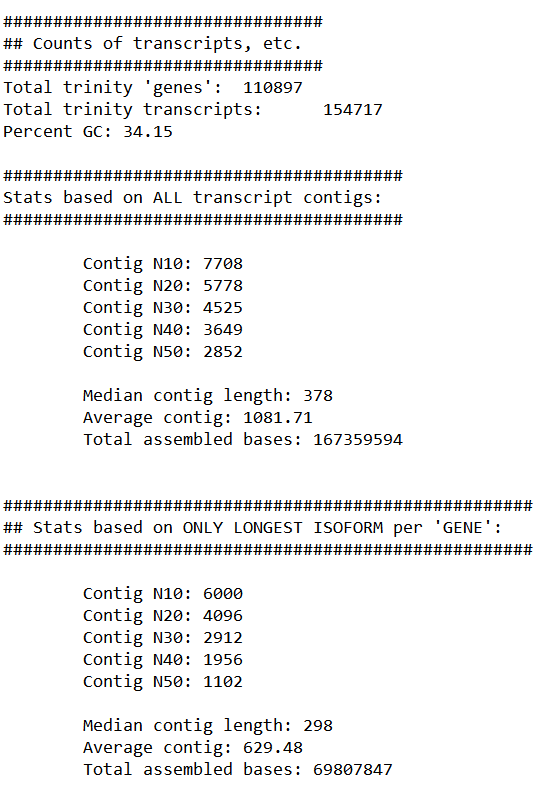

Supplement: Supplementary Data 2 — Transcriptome data information. [file Image_1.PNG]
